# Population-Based COVID-19 Screening in Mexico: Assessment of Symptoms and Their Weighting in Predicting SARS-CoV-2 Infection

**DOI:** 10.3390/medicina57040363

**Published:** 2021-04-08

**Authors:** Margarita L Martinez-Fierro, Martha Diaz-Lozano, Claudia Alvarez-Zuñiga, Leticia A Ramirez-Hernandez, Roxana Araujo-Espino, Perla M Trejo-Ortiz, Fabiana E Mollinedo-Montaño, Yolanda Ortiz-Castro, Sodel Vazquez-Reyes, Perla Velasco-Elizondo, Lidia Garcia-Esquivel, Arturo Araujo-Conejo, Idalia Garza-Veloz

**Affiliations:** 1 Molecular Medicine Laboratory, Unidad Academica de Medicina Humana y C.S, Universidad Autónoma de Zacatecas, Zacatecas 98160, Mexico; 08diazmartha@gmail.com (M.D.-L.); cldaalzu30798@gmail.com (C.A.-Z.); yolandaortiz.castro@gmail.com (Y.O.-C.); 2 Unidad Academica de Matematicas, Universidad Autonoma de Zacatecas, Zacatecas 98160, Mexico; lramirez@uaz.edu.mx; 3 Unidad Academica de Enfermería, Universidad Autónoma de Zacatecas, Zacatecas 98160, Mexico; araujo_navar@hotmail.com (R.A.-E.); perlu11@yahoo.com.mx (P.M.T.-O.); fabiana.mollinedo@uaz.edu.mx (F.E.M.-M.); 4 Unidad Academica de Ingenieria Eléctrica, Universidad Autónoma de Zacatecas, Zacatecas 98160, Mexico; vazquezs@uaz.edu.mx (S.V.-R.); pvelasco@uaz.edu.mx (P.V.-E.); 5 Clinica Universitaria, Unidad Academica de Medicina Humana y C.S, Universidad Autónoma de Zacatecas, Zacatecas 98160, Mexico; lidia.garcia.e11@hotmail.com; 6 Hospital General Zacatecas “Luz González Cosío”, Servicios de Salud de Zacatecas, Zacatecas 98160, Mexico; aaraujo2002@yahoo.com

**Keywords:** SARS-CoV-2, COVID-19, symptoms

## Abstract

*Background and Objectives*: Sentinel surveillance in the early stage of the COVID-19 pandemic in Mexico represented a significant cost reduction and was useful in estimating the population infected with SARS-CoV-2. However, it also implied that many patients were not screened and therefore had no accurate diagnosis. In this study, we carried out a population-based SARS-CoV-2 screening in Mexico to evaluate the COVID-19-related symptoms and their weighting in predicting SARS-CoV-2 infection. We also discuss this data in the context of the operational definition of suspected cases of COVID-19 established by the Mexican Health Authority’s consensus. *Materials and Methods*: One thousand two hundred seventy-nine subjects were included. They were screened for SARS-CoV-2 using RT-PCR. The weighting of COVID-19 symptoms in predicting SARS-CoV-2 infection was evaluated statistically. *Results*: Three hundred and twenty-five patients were positive for SARS-CoV-2 and 954 were negative. Fever, asthenia, dysgeusia, and oxygen saturation predicted SARS-CoV-2 infection (odds ratios ranged from 1.74 to 4.98; *p* < 0.05). The percentage of asymptomatic COVID-19 patients was 36% and only 38.15% met the Mexican operational definition. Cq-values for the gene N of SARS-CoV-2 were significantly higher in asymptomatic subjects than in the groups of COVID-19 patients with neurological, respiratory, and/or musculoskeletal manifestations (*p* < 0.05). *Conclusions*: Dysgeusia, fever, and asthenia increased the odds of a positive result for COVID-19 1.74–4.98-fold among the study population. Patients with neurological, respiratory, and/or musculoskeletal manifestations had higher viral loads at COVID-19 diagnosis than those observed in asymptomatic patients. A high percentage of the participants in the study (61.85%) did not meet the operational definition for a suspected case of COVID-19 established by the Mexican Health Authority’s consensus, representing a high percentage of the population that could have remained without a COVID-19 diagnosis, so becoming a potential source of virus spread.

## 1. Introduction

The recent spread of severe acute respiratory syndrome coronavirus 2 (SARS-CoV-2) and its associated coronavirus disease (COVID-19) has gripped the entire international community and caused widespread public health concerns [1]. Worldwide, until 27 March 2021, there were 126,409,918 cases confirmed and 2,771,414 deaths caused by COVID-19 [2]. The first case reported in Mexico was on 28 February 2020 [3] and until now, a total of 2,224,904 confirmed cases and 201,429 deaths has been attributed to COVID-19. 

According to previous data, the tendency of the spread of SARS-CoV-2 largely followed an exponential growth, and the mean basic reproduction number (R0) for COVID-19 was estimated to range from 2.24 to 3.58, associated with a two- to eight-fold increase in the reported rate of COVID-19 [4]. The transmission of SARS-CoV-2 is primarily dependent on various routes of human-to-human transmission that include direct contact with the aerial droplets released during the conversation, coughing, and sneezing by infected persons [5]. The most common symptoms of COVID-19 are fever, fatigue, dry cough, dyspnea, and malaise [6,7,8,9]. Less common symptoms include sputum production, headache, hemoptysis, diarrhea, anorexia, sore throat, chest pain, chills, nausea, and vomiting. The clinical manifestation of COVID-19 is quite variable and is related to the age of the patient and comorbidities. In a general manner, older men (>60 years old) with comorbidities are more likely to develop a severe form of COVID-19, whereas most young people and children present only the mild forms of disease or they are asymptomatic [6].

Due to the rapid increase in SARS-CoV-2 infected cases, global governments and healthcare institutions, such as the World Health Organization (WHO), have made important efforts to face the COVID-19 pandemic. These efforts have focused mainly on containing the disease, which led to adopting pandemic preparedness activities and proactive approaches [10]. One of the most remarkable measures applied by the member countries of the WHO was the implementation of different types of epidemiological surveillance and the combination of surveillance systems and/or the adaptation of existing surveillance systems, all based on the conditions extant in each country [11]. By definition, surveillance is the ongoing systematic collection, analysis, interpretation, and dissemination of data regarding a health-related event for determining actions [12]. Specifically, in COVID-19, surveillance involves monitoring the spread of the disease to identify patterns of progression and the application of preventive and control measures [12]. The Government of Mexico implemented the Sentinel surveillance, which consisted of a network of 475 healthcare providers and/or hospitals distributed throughout the national territory, which were recruited to regularly report data about the disease. These units had a high probability of seeing the cases of COVID-19, having good laboratory facilities and qualified staff [13]. Surveillance in Mexico involved those individuals who showed signs and symptoms of COVID-19 being able to access evaluation and SARS-CoV-2 testing, as long as they met the operational definition for suspected COVID-19 cases established by the Mexican health authorities. Until 24 August, these criteria included meeting two out of three of the following symptoms: fever ≥ 38 °C and dry cough and/or headache, in addition to other COVID-19-related symptoms and the presence of comorbidity [14,15,16]. Although for COVID-19, Sentinel surveillance represented a significant cost reduction and was useful for estimating the population affected by COVID-19, it implied that many patients were not screened and therefore had no accurate diagnosis because they did not meet the operational definition [3,12]. According to the above, reports related to COVID-19 in Mexico must be analyzed in the context of the operational definition in force at that time to avoid bias in analysis and interpretation. In this study, we performed a population-based SARS-CoV-2 screening in the early stage of the pandemic in Mexico to evaluate the symptoms of COVID-19 and their weighting to predict SARS-CoV-2 infection. The comparison of the frequency of symptoms according to the reported data in the context of the operational definition was also considered to obtain a more realistic observation of the frequency of the symptoms manifested in Mexican COVID-19 patients.

## 2. Materials and Methods

### 2.1. Patients and Study Definitions

**Figure 1:**
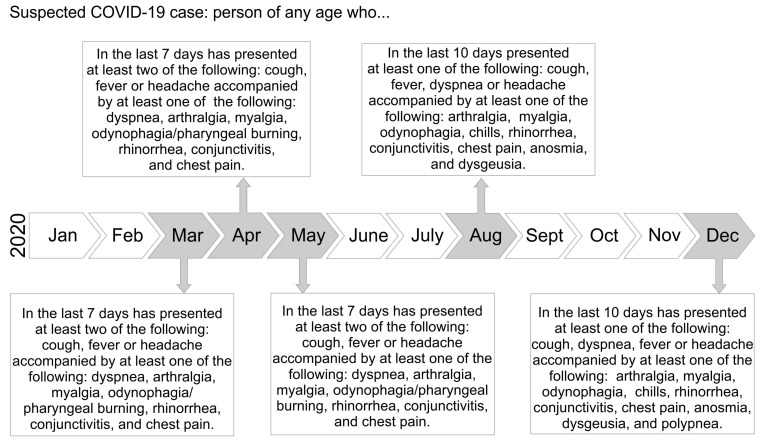
Evolution of the official Mexican operational definition of COVID-19 suspicious case. The Mexican government has updated the official operational definition of a suspected COVID-19 case five times throughout 2020. The operational definition from March to May 2020 included: a person of any age who had filed in the previous 7 days at least two of the following signs and symptoms: cough, fever, or headache, accompanied by at least one of the following signs and symptoms: dyspnea, arthralgia, myalgia, odynophagia, rhinorrhea, conjunctivitis, and chest pain. In August, the update was based on increasing to 10 the days in which the symptoms could occur, in addition, it decreased to one symptom presented in 10 days, and the symptoms were divided into major (cough, fever, dyspnea, or headache) and minor (myalgia, arthralgia, odynophagia, chills, chest pain, rhinorrhea, anosmia, dysgeusia, conjunctivitis). The last update was in December 2020 and included polypnea as a minor symptom [9].

This was a cross-sectional study. The Ethics and Research Committees of the Academic Unit of Human Medicine and Health Sciences from the Universidad Autonoma de Zacatecas and the Alpha Medical Center approved this cross-sectional study carried out in Zacatecas, Mexico from April to August 2020 (Approval ID: AMCCI-FSARSC2-006 and 007). Detailed information related to the protocol was provided to the participants and written informed consent was obtained. There were no exclusion criteria for the study. A total of 1229 patients were included: 325 were positive for SARS-CoV-2 (COVID-19 cases) and 954 were negative (controls). The patient recruitment was carried out in the Molecular Medicine Laboratory from the Academic Unit of Human Medicine and Health Sciences at the Universidad Autonoma de Zacatecas in Zacatecas, Mexico. All the participants who provided signed informed consent provided a biological sample for SARS-CoV-2 screening and completed a questionnaire concerning the risk factors, demographic and clinical data, and signs and symptoms related to COVID-19 [13]. The Mexican government modified the operational definition of a suspected COVID-19 case five times during 2020 (Figure 1).

For this protocol, a suspected case of COVID-19 considered the operational definition in force until 24 August 2020. This included a patient who met two out of three of the following symptoms: fever ≥ 38 °C, dry cough and/or headache, and having at least one other COVID-19 related symptoms (asthenia, odynophagia, myalgia, arthralgia, rhinorrhea, conjunctivitis, anosmia, dysgeusia, nausea, abdominal pain, and diarrhea) and underlying risk conditions (pregnancy, immunosuppression, previous lung disease, diabetes mellitus, systemic arterial hypertension, adults >65 years old, or obesity) [14,15,16]. Is important to note that the official Mexican operational definition of a suspected COVID-19 case was not taken as an exclusion criterion for the patients to be sampled; patients requested the screening for SARS-CoV-2 by personal choice.

### 2.2. Biological Samples and SARS-CoV-2 Screening

Nasopharyngeal and oropharyngeal swab samples were obtained from each participant; subsequently, they were packed and transported in triple packaging at a temperature of 4 °C following the guidelines of the WHO and the Pan-American Health Organization for the handling and transport of viral SARS-CoV-2 specimens [17,18]. The samples were sent to the Molecular Medicine Laboratory of the Academic Unit of Human Medicine and Health Sciences of the Autonomous University of Zacatecas for processing [16]. Exudate samples were screened for SARS-CoV-2 with a one-step RT-PCR assay using the CDC real-time RT-PCR panel (Integrated DNA Technologies, Coralville, IA, USA). SARS-CoV-2 detection was analyzed in a Step One Plus Real-Time PCR system (Thermo Fisher Scientific, Waltham, MA, USA) and interpreted according to the manufacturer’s instructions.

### 2.3. Data Analysis

General findings of the study population were represented as the mean ± standard deviation (SD) and percentages. Comparisons of the risk factors and the clinical findings among the groups were performed using a Chi-Square or Fisher exact test for categorical variables and a *t*-test or Mann–Whitney U test for continuous variables. Differences between Cq values between patients grouped by symptoms were evaluated using the Kruskal-Wallis analysis of variance (ANOVA) on ranks and Dunn’s method as a multiple comparison procedure. The odds ratios (ORs) with Yates continuity correction were calculated for significant comparisons. Multivariate logistic regression was used to correct the risk values using the SARS-CoV-2 qRT-PCR status as the dependent variable. Analysis of correspondence was done to explore relationships among categorical variables. Statistical analysis was carried out with the SigmaPlot v12.0 (Systat Software Inc., San Jose, CA, USA) and STATISTICA v12.0 (StatSoft Inc.) software. A *p*-value of <0.05 was considered significant.

## 3. Results

One thousand two hundred seventy-nine patients participated in this study: 325 of them showed positive results for SARS-CoV-2 (COVID-19 cases) and 954 showed negative results (controls). Men made up 66.15% of the COVID-19 cases and 59.5% of the controls (*p* = 0.039). The mean age in the COVID-19 group was 40.95 ± 14.48 years, whereas in controls it was 37.76 ± 13.25 years (*p* < 0.05). The most frequent comorbidity in the study population was obesity (Table 1); it was observed in 28% of the patients with COVID-19 and 21.8% of the controls (*p* = 0.028). Type 2 diabetes mellitus (T2DM) was presented in 25 (7.69%) COVID-19 cases and in 30 (3.17%) of the control group (*p* < 0.001). Mean oxygen saturation was 92.65 (±3.28) and 93.46 (±2.09) in the subjects with and without SARS-CoV-2 infection, respectively (*p* < 0.050). There were no differences between study groups regarding the presence of arterial hypertension, chronic obstructive pulmonary disease (COPD), chronic kidney disease, or asthma (*p* > 0.05).

Of the COVID-19 cases, the percentage of asymptomatic patients was 36%, and the percentage with only one symptom was 6.46%. In the same sense, of the total number of patients with a SARS-CoV-2-positive result, only 38.15% met the Mexican operational definition. 

Table 2 displays a summary of the main symptoms observed in the study population. 

Univariate analysis showed that, of the 21 symptoms evaluated, 17 showed differences between COVID-19-positive cases and controls (*p* < 0.05). The most frequent symptoms in COVID-19 cases were dry cough (35.38%), headache (32.92%), and fever (30.15%). In the control group, headache (19.47%) and odynophagia (15.45%) remained the most frequent symptoms observed. The ORs calculated ranged from 1.9 (for diarrhea and odynophagia) to 8.5 (for dysgeusia). In univariate analysis, there were no statistical differences in the proportions of polypnea, vomiting, conjunctivitis, and convulsions between the COVID-19 cases and the controls (*p* > 0.05).

After statistical correction, fever, asthenia, dysgeusia, and oxygen saturation were the variables that remained with significant *p*-values in the multivariate analysis (Table 3). Patients with dysgeusia had a 4.98-fold higher risk of being positive for SARS-CoV-2 (*p* = 0.005; 95% CI: 1.6–15.1). In the same way, having a fever increased the odds of a positive result for COVID-19 by 2.087 times in the study population (*p* = 0.004; 95% CI: 1.3–3.4).

Oxygen saturation was the only continuous variable with prediction value for a positive/negative result for COVID-19 among the study population (*p* = 0.003; OR = 0.908; 95% CI: 0.852–0.967).

**Figure 2:**
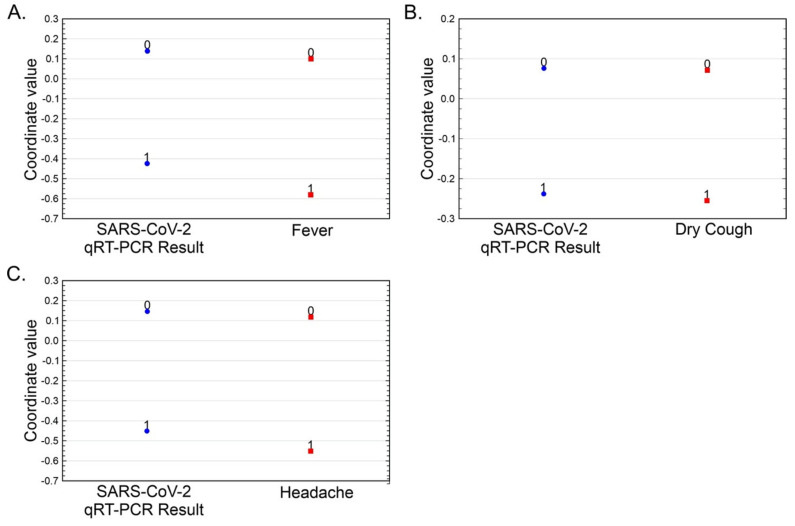
Correspondence analysis of SARS-CoV-2 qRT-PCR results and the symptoms with the stronger predictive value. Tables of 2 × 2 from SARS-CoV-2 qRT-PCR results. Each symptom is used as input and represented as a one-dimensional plot. The three symptoms with the strongest SARS-CoV-2 qRT-PCR predictive value (*p* < 0.001) are displayed in the figure: (**A**) fever, (**B**) dry cough, and (**C**) headache. The Eigenvalues calculated with 100% inertia were 0.05963 for fever, 0.01945 for dry cough, and 0.06573 for headache.

To identify whether there was some bias in the analysis because of differences in the number of participants between groups, a correspondence analysis was carried out. The results of this data modeling are shown in Table 4 and Figure 2A–C. Correspondence analysis is a multivariate graphical technique designed to explore relationships among categorical variables and allows comparison between row or column labels based on distances between points representing the variables. The chi-square test of independence is used to determine whether the association between two categorical variables is significant. If we obtain a significant chi-square value (*p* < 0.05), we conclude that the two variables in question are indeed related. 

The correspondence analysis revealed that only fever (*p* < 0.001), dry cough (*p* < 0.001), and headache (*p* < 0.001) had strong associations with a positive SARS-CoV-2 qRT-PCR result. It also showed that the absence of these symptoms was related to a negative SARS-CoV-2 qRT-PCR result. Multivariate analysis did not show any strong relationship between asthenia, odynophagia, myalgia, arthralgia, rhinorrhea, conjunctivitis, anosmia, dysgeusia, nausea, abdominal pain, nor diarrhea, and the SARS-CoV-2 qRT-PCR result in any of three groups evaluated (*p* > 0.05).

**Figure 3:**
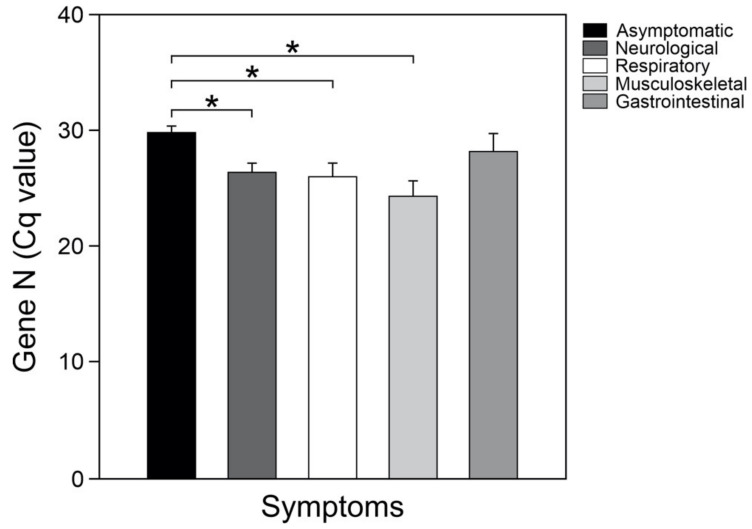
Values of Cq of SARS-CoV-2 positive patients classified by a group of COVID-19-related symptoms. * *p* < 0.05.

Finally, to evaluate whether there was a relationship between the Cq-values obtained from the SARS-CoV-2 screening at the time of diagnosis and the presence or absence of specific manifestations, the COVID-19 patients were subclassified according to the origin of their most predominant COVID-19-related symptoms. In accordance with the above, the patients were grouped as: those subjects with neurological (fever, headache, chills, anosmia, dysgeusia, irritability), respiratory (cough, dyspnea, chest pain, rhinorrhea), musculoskeletal (myalgia, arthralgia, asthenia, cyanosis), and gastrointestinal (diarrhea, odynophagia, vomiting, abdominal pain) symptoms, and a group of asymptomatic participants. Considering the asymptomatic patients as a reference, the mean Cq-values for the gene N of SARS-CoV-2 were significantly lower (Figure 3) in the groups of patients with neurological, respiratory, and/or musculoskeletal manifestations (*p* < 0.05).

## 4. Discussion

In this study, we performed a population-based SARS-CoV-2 screening during phase 3 of the COVID-19 pandemic in Mexico to evaluate the COVID-19 symptoms and their weighting in predicting SARS-CoV-2 infection. The symptoms were then grouped to compare their association with the viral load of the COVID-19 patients. A comparison of the frequency of symptoms was also considered to obtain a realistic description of the symptoms manifested by Mexican COVID-19 outpatients. All participants in our protocol were had no severity data at the time of diagnosis. Our results showed that the most common symptoms observed in patients with COVID-19 were dry cough, fever, and headache, these being present in up to 35.38% (dry cough) of the SARS-CoV-2-positive patients. After statistical correction, fever, asthenia, and oxygen saturation were the most significant variables to predict SARS-CoV-2 infection. In this study, patients who had dysgeusia, fever, or asthenia showed a 4.98-, 2.1-, and 1.7-fold higher risk of a positive result for SARS-CoV-2 infection, respectively. These results represent the first symptom weighting in a population-based screening in Mexico with no exclusion criteria and limitations established by the operational definition established in the Mexican health guidelines. In the same way, differences in the proportions of COVID-19-related symptoms between other studies and ours (Table 5) [18,19,20], indicated the importance of expanding the range of symptoms and maintaining updated over time the operational definition in the Mexican health surveillance system. 

Asymptomatic individuals represent a substantial fraction of the population infected with SARS-CoV-2 and is estimated at 17.9% representing a potential source of virus spread [16]. Our data showed that the percentage of asymptomatic COVID-19 patients was 36%, this being a higher percentage than the 14.7% previously reported [16]. On the other hand, of the total number of patients with a positive SARS-CoV-2 result, only 38.15% met the operational definition for a suspected case of COVID-19 established by the Mexican Health Authority’s consensus. This percentage was also higher than that reported in a previous study carried out by our group (from June to July 2020), in which the close contacts of COVID-19 cases were screened and the percentage of those close contacts with a SARS-CoV-2 positive result that met the established operational definition was only 17.6% [16]. These results together indicated that in our city (Zacatecas, Mexico), in the period evaluated (March to August 2020), of the total number of participants with COVID-19 screened, between 61.85% and 82.4% would not have been able to access a SARS-CoV-2 test in a public hospital, so representing a high percentage of the population that could have stayed without a COVID-19 diagnosis. Even though our sampled population did not include people from all cities in the country, these percentages may still represent an approximation of the population of Mexico not evaluated in that period under the Sentinel model.

Interestingly, grouping the symptoms according to their origin and considering the asymptomatic patients as a reference, the mean SARS-CoV-2 viral load was significantly higher in the groups of patients with neurological, respiratory, and/or musculoskeletal manifestations; being highest in the group with musculoskeletal symptoms. Previous studies have suggested that recovered patients from COVID–19 experienced a biphasic disease: the first phase (which lasts 7–10 days) was related to worsening of clinical and radiological symptoms associated with intense virus replication. The second phase would have featured clinical and radiological improvement, accompanying reduction of viremia [30]. Viral load together with hematological signs and symptoms may also provide clues to aid diagnosis and/or prognosis of the disease [30,31]. Unfortunately, in our study, additional clinical parameters of COVID-19 severity were not available. Additional studies are needed to investigate the modified variables (genetics of the people, viral variants, among others) that may explain the relationship between the viral load and the origin of the symptoms observed in our study and how the hematological features of the patient could be modified.

## 5. Conclusions

Dysgeusia, fever, and asthenia increased the odds of a positive result for COVID-19 by 1.74–4.98-fold among the studied population. Patients with neurological, respiratory, and/or musculoskeletal manifestations had higher viral load at COVID-19 diagnosis than that observed in asymptomatic patients. A high percentage of the participants in the study (61.85%) did not meet the operational definition for a suspected case of COVID-19 established by the Mexican Health Authority’s consensus, representing a high percentage of the population that could have remained without a COVID-19 diagnosis. These results indicate the importance of expanding the range of symptoms in the operational definition and of increasing the number of SARS-CoV-2 tests to avoid the spread of infection and disease.

## Figures and Tables

**Table 1:** Main findings observed in the study population categorized by COVID-19 cases and controls.

Comorbidity/Risk Factor	COVID-19 Cases (*n* = 325)	Controls (*n* = 954)	*p*-Value	Odds Ratio	95% CI
Type 2 diabetes mellitus	25 (7.69)	30 (3.17)	<0.001 *	2.5	1.471–4.390
Obesity	91 (28)	206 (21.79)	0.028 *	1.3	1.047–1.859
Smoking	60 (18.46)	207 (21.90)	0.217	0.8	0.586–1.111
Hypertension	35 (10.76)	81 (8.57)	0.282	1.3	0.847–1.956
Endocrinological disorders ^1^	6 (1.84)	29 (3.04)	0.346	0.6	0.247–1.458
Allergies	5 (1.53)	23 (2.41)	0.478	0.6	0.238–1.677
Rheumatic diseases	4 (1.23)	18 (1.88)	0.59	0.6	0.218–1.929
Asthma	8 (2.46)	17 (1.79)	0.61	1.4	0.589–3.223
COPD ^2^	2 (0.61)	93 (0.31)	0.821	1.9	0.323–11.688
Cardiovascular disease	3 (0.92)	11 (1.16)	0.959	0.7	0.219–2.853
Chronic kidney disease	1 (0.30)	5 (0.52)	0.973	0.5	0.0675–4.985

Data are represented as frequency and percentages. ^1^ Hypothyroidism, hyperthyroidism, and polycystic ovary syndrome; ^2^ Chronic obstructive pulmonary disease * *p* < 0.05.

**Table 2:** Comparison of symptoms between groups of patients with and without SARS-CoV-2 infection.

Symptoms (*n*, %)	COVID-19 Cases (*n* = 325)	Controls (*n* = 954)	*p*-Value	Odds Ratio	95% CI
Fever	98 (30.15)	95 (10.05)	<0.001 *	3.8	2.811–5.308
Dry cough	115 (35.38)	119 (12.59)	<0.001 *	3.8	2.822–5.119
Headache	107 (32.92)	184 (19.47)	<0.001 *	2.03	1.531–2.691
Chills	39 (12.0)	46 (4.84)	<0.001 *	2.67	1.712–4.185
Odynophagia	85 (26.15)	146 (15.45)	<0.001 *	1.9	1.430–2.626
Myalgia	68 (20.92)	87 (9.20)	<0.001 *	2.6	1.845–3.690
Arthralgia	55 (16.92)	65 (6.87)	<0.001 *	2.7	1.878–4.049
Asthenia	67 (20.61)	90 (9.52)	<0.001 *	2.4	1.746–3.485
Anosmia	59 (18.15)	29 (3.05)	<0.001 *	7.03	4.420–11.202
Dysgeusia	61 (18.76)	25 (2.63)	<0.001 *	8.5	5.257–13.872
Chest pain	35 (10.76)	50 (5.29)	0.001 *	2.1	1.375–3.394
Irritability	14 (4.30)	12 (1.26)	0.002 *	3.5	1.609–7.680
Rhinorrhea	38 (11.62)	58 (6.11)	0.002 *	2.03	1.323–3.127
Diarrhea	35 (10.76)	55 (5.67)	0.004 *	1.9	1.258–3.058
Dyspnea	29 (8.92)	43 (4.45)	0.005 *	2.05	1.260–3.351
Abdominal pain	26 (8.0)	37 (3.91)	0.005 *	2.1	1.271–3.583
Cyanosis	11 (3.38)	10 (1.05)	0.01 *	3.2	1.378–7.786
Polypnea	6 (1.84)	7 (0.73)	0.163	2.5	0.844–7.587
Vomit	8 (2.46)	14 (1.48)	0.357	1.6	0.698–4.038
Conjunctivitis	14 (4.30)	30 (3.16)	0.423	1.3	0.722–2.634
Convulsion	2 (0.61)	5 (0.52)	0.8	1.1	0.225–6.029

Data are represented as frequency and percentages. *p*-values were obtained by the comparison of symptoms between patients with SARS-CoV-2 RT-PCR (+) and RT-PCR (-). * Significant *p*-values of <0.05. The odds ratio is the comparison between the proportions of cases positive and negative for SARS-CoV-2 by qRT-PCR.

**Table 3:** Variable modeling by multivariate logistic regression analysis.

Variable	Coefficient	Standard Error	Wald Statistic	*p*-Value	Odds Ratio	95% CI
Oxygen saturation	−0.0964	0.0323	8.938	0.003 *	0.908	0.852–0.967
Fever	0.736	0.253	8.472	0.004 *	2.087	1.272–3.424
Dysgeusia	1.605	0.566	8.033	0.005 *	4.976	1.641–15.094
Asthenia	0.555	0.269	4.237	0.040 *	1.741	1.027–2.952
Irritability	1.119	0.687	2.654	0.103	3.063	0.797–11.774
Dyspnea	−0.636	0.397	2.569	0.109	0.529	0.243–1.152
T2DM	0.430	0.354	1.475	0.225	1.537	0.768–3.074
Obesity	0.181	0.171	1.119	0.290	1.198	0.857–1.676
Sex	−0.156	0.164	0.900	0.343	0.856	0.620–1.181
Age	0.0055	0.00609	0.818	0.366	1.006	0.994–1.018
Diarrhea	0.234	0.330	0.502	0.479	1.264	0.661–2.415
Arthralgia	0.249	0.393	0.403	0.526	1.283	0.594–2.770
Rhinorrhea	0.204	0.323	0.401	0.527	1.227	0.652–2.309
Abdominal pain	−0.197	0.394	0.250	0.617	0.821	0.379–1.778
Headache	0.110	0.235	0.219	0.640	1.116	0.705–1.768
Chills	0.153	0.349	0.191	0.662	1.165	0.588–2.308
Myalgia	0.104	0.362	0.0825	0.774	1.109	0.546–2.254
Dry cough	−0.000002	0.000009	0.0472	0.828	1.000	1.000–1.000
Anosmia	0.0861	0.565	0.0232	0.879	1.090	0.360–3.301
Odynophagia	0.0220	0.221	0.0099	0.921	1.022	0.663–1.576
Chest pain	0.0250	0.340	0.005	0.941	1.025	0.527–1.995
Cyanosis	−0.0195	0.640	0.0009	0.976	0.981	0.280–3.437

Odds ratio obtained from multivariate regression analysis; T2DM: type 2 diabetes mellitus. * *p*-value < 0.05.

**Table 4:** Correspondence analysis for symptoms with significant *p*-values.

Variable	qRT-PCR for SARS-CoV-2		Eigen Value	Chi-Square	Degrees of Freedom	*p*-Value
	Positive	Negative				
Fever (%)	7.72	17.89	0.05963	75.67	1	<0.001
Dry Cough (%)	9.06	16.55	0.01945	83.41	1	<0.001
Headache (%)	8.43	17.18	0.06573	24.68	1	<0.001

**Table 5:** Comparison of COVID-19-related symptoms.

Variable/Study	MEX This Study	MEX [21]	CHN [22]	CHN [23]	CHN [24]	DEU [19]	MEX [16]	MEX [25]	MEX [26]	CHN [27]	BGR [8]	CHN [28]	BEL [29]
COVID-19 cases	325	16	41	1099	99	26	34	196,738	164	138	138	140	1420
Age, mean (years)	40.9	47.8	49.0	47.0	55.5	52.4	-	36	57.3	56	52.9	57	39.17
Sex (male)	215 (66.1)	8 (50)	30 (73)	640 (58.1)	67 (68)	14 (35)	-	244,171	114 (69.5)	75 (54.3)	87 (63)	71(50.7)	458 (32.3)
Fever	98 (30.15)	43.80	40 (98)	473 (43.8)	82 (83)	9 (34.6)	5 (14.7)	60,209 (56.7)	138 (84.1)	136 (98.6)	100 (138)	110/120 (91.7)	645 (45.4)
Dry cough	115 (35.38)	68.80	31 (76)	745 (67.8)	81 (82)	19 (73.1)	10 (29.4)	60,720 (55.4)	131 (79.8)	82 (59.4)	95 (68.8)	90/120 (75.0)	897 (63.2)
Headache	107 (32.92)	81.30	3 (8)	150 (13.6)	8 (8)	3 (11.5)	13 (38.2)	106,103 (44.4)		9 (6.5)	95 (68.8)	-	998 (70.3)
Dyspnea	29 (8.92)	43.80	22 (55)	205 (18.7)	31 (31)	4 (15.4)	6 (17.6)	34,588 (49.8)	152 (92.6)	43 (31.2)	39 (28.52)	44/120 (36.7)	697 (49.1)
Irritability	14 (4.30)	-	-	-	-	-	6 (17.6)	-			-	-	-
Diarrhea	35 (10.76)	-	1 (3)	42 (3.8)	2 (2)	1 (3.8)	6 (17.6)	32,843 (45.7)	29 (17.6)	14 (10.1)	7 (5.0)	18/139 (12.9)	473 (38.1)
Chest pain	35 (10.76)	31.30	-	-	2 (2)		9 (26.4)	-	-	-	-	-	173 (27.2) *
Chills	286 (88)	-	-	126 (11.5)	-	5 (19.5)	4 (11.7)	39,003 (56.1)	-	-	-	-	-
Odynophagia	85 (26.15)	-	-	-	-		12 (35.2)	-	-	24 (17.4)	46 (33.3)	-	274 (19.3)
Myalgia	68 (20.92)	62.50	18 (44)	164 (14.9)	11 (11)	7 (26.9)	13 (38.2)	93,926 (50.9)	84 (51.2)	48 (34.8)	67 (48.5)	-	887 (62.5)
Arthralgia	55 (16.92)	62.50	-	with myalgia	-		9 (26.4)	with myalgia	-		With myalgia	-	519 (36.5)
Asthenia	67 (20.61)	-	-	419 (38.1)	-	5 (19.2)	7 (20.5)	-	-	96 (69.6)	124 (89.8)	90/120 (75.0)	514 (63.3) *
Rhinorrhea	38 (11.62)	50	-	-	4 (4)	5 (19.2)	10 (29.4)	48,082 (51.4)	31 (18.9)	-	-	-	854 (60.1)
Polypnea	6 (1.84)	-	-	-		-	3 (8.8)	-	-	-	-	-	-
Vomit	8 (2.46)	-	-	55 (5.0)	1 (1)	0 (0)	0 (0.0)	15,841 (49.8)	-	5 (3.6)	-	7/139 (5.0)	272 (19.2) and nausea
Abdominal pain	26 (8.0)	-	-	-	-	0 (0)	1 (2.9)	17,515 (44.8)	-	3 (2.2)	-	8/139 (5.8)	270 (19.1)
Conjunctivitis	14 (4.30)	6.30	-	-	-	-	5 (14.7)	13,738 (46.6)	-	-	12 (8.6)	-	644 (45.4)
Anosmia	59 (18.15)	37.50	-	9 (0.8)	-	7 (26.9)	5 (14.7)	-	-	-	-	-	997 (70.2)
Dysgeusia	61 (18.76)	37.50	-	-	-	-	3 (.8)	-	-	-	-	-	770 (54.2)
Cyanosis	11 (3.38)	-	-	-	-	-	0 (0.0)	-	-	-	-	-	-
Convulsions	2 (0.61)	-	-	-	-	-	-	-	-	-	-	-	-

-: No data; * Some data were not available, and therefore, the proportion was calculated on a reduced sample. MEX: Mexico; CHN: China; DEU: Germany; BGR: Bulgaria; BEL: Belgium.

## Data Availability

Additional data that support the findings of this study are available from the corresponding author [M.L.M.-F.] and [I.G.-V.], upon reasonable request.
